# A novel efficient β-glucanase from a paddy soil microbial metagenome with versatile activities

**DOI:** 10.1186/s13068-016-0449-6

**Published:** 2016-02-13

**Authors:** Yu Zhou, Xu Wang, Wei Wei, Jimin Xu, Wei Wang, Zhongwen Xie, Zhengzhu Zhang, Hongchen Jiang, Qi Wang, Chaoling Wei

**Affiliations:** State Key Laboratory of Tea Biology and Utilization, School of Tea and Food Science Technology, Anhui Agricultural University, Hefei, 230036 China; Institute of Quality and Standard for Agro-products, Zhejiang Academy of Agricultural Sciences, Hangzhou, 310021 China; State Key Laboratory of Biogeology and Environmental Geology, China University of Geosciences, Wuhan, 430074 China; Novus International (Shanghai) Inc, Shanghai, 200080 China

**Keywords:** Metagenomic library, Versatile β-glucanase, Glycoside hydrolase family 9, Transglycosylation, Endoglucanase, Exoglucanases

## Abstract

**Background:**

Cellulose, an abundant and renewable polysaccharides, constitutes the largest resource for bioconversion of biofuels. Plant polysaccharides hydrolysis is catalyzed by cellulases, which include endoglucanases, exoglucanases, and β-glucosidases. Converting cellulose and hemicellulose to short chains of oligosaccharides by endo-/exoglucanases is the key step for biofuel transformation. Intriguingly, β-glucanases with transglycosylation activity not only can relieve product inhibition of glucan hydrolysis but also has potential application as biocatalysts for functional materials.

**Results:**

Here, a metagenomic fosmid library was constructed from a paddy soil for cellulase screening. One purified clone showing carboxymethylcellulase activity was isolated, and the complete β-glucanase gene (*umcel9y*-*1*) was cloned and overexpressed in *Escherichia coli*. Phylogenetic analysis indicated that β-glucanase Umcel9y-1 belonged to the theme C of glycoside hydrolase family 9. Amino acids sequence showed 58.4 % similarity between Umcel9y-1 and its closest characterized reference, cellulase Cel01. Biological characterization showed that Umcel9y-1 was an efficient endoglucanase and also exhibited high activities of exoglucanase and transglycosylation. The transglycosylation products of Umcel9y-1 including sophorose, laminaribiose, and gentiobiose, and transglycosylation was detected under all activated conditions. The order of catalytic efficiency for polysaccharides, cellooligosaccharides, and aryl-β-glycosides was p-nitrophenol-D-cellobioside, barley glucan, cellopentaose, cellotetraose, cellotriose, hydroxyethylcellulose, cellohexose, laminarin, and carboxymethylcellulose, respectively. The barley glucan was the optimal polysaccharides for Umcel9y-1 with *K*_m_ and *K*_cat_/*K*_m_ values of 13.700 mM and 239.152 s^−1^ mM^−1^, respectively.

**Conclusion:**

Biological characterizations of recombinant Umcel9y-1 showed that the versatile β-glucanase had efficient endoglucanase activity to barley glucan and also exhibited high activities of exoglucanase and transglycosylation. The optimum conditions of recombinant Umcel9y-1 was pH 6.5–7.0 at 37 °C with predominant halotolerance and high-thermal stability. These results indicate that the novel metagenomic-derived β-glucanase may be a potent candidate for industrial applications.

**Electronic supplementary material:**

The online version of this article (doi:10.1186/s13068-016-0449-6) contains supplementary material, which is available to authorized users.

## Background

Cellulose fibers are composed of bundles of linear polymers of D-glucose linked by β-1, 4-glycosyl bonds [1]. Cellulose is the most abundant and renewable polysaccharide and constitutes one-third of all existing plant cell-wall material, which is the largest potential resource for bioconversion of ethanol and other biofuels, feedstock chemicals, and pharmaceuticals [2–4]. Cellulases catalyze cellulolysis through hydrolyzing β-1, 4-glycosidic bonds in cellulose, and mainly consisted of three types of enzymes: endoglucanases (EC3.2.1.4), exoglucanase (or cellobiohydrolase, EC3.2.1.91), and β-glucosidase (EC3.2.1.21) [5]. Endoglucanases randomly hydrolyze internal β-1, 4-glycosidic bonds to decrease the length of cellulose chain, exoglucanases split off cellobiose from the shortened cellulose chains, and β-glucosidases degrade cellobiose to glucose [5].

Nowadays, some cellulases from fungi and bacteria have been technically employed in cellulose biotransformation, including polysaccharide degradation, textile processing, juice and wine fermentation, animal feed and fuel production, and other industries [6–8]. Recently, cellulases (including β-glucanase) with transglycosylation activity are attracted as substitute of glycosyltransferases for the synthesis of stereo- and regiospecific glycosides (or oligosaccharides) which are considered as functional materials, nutraceuticals, or pharmaceuticals [9, 10]. Previously, glycosyltransferases were the primary choice as the biocatalyst for glycosidic synthesis, but the industrial application processes come to a standstill for years. The major difficulties are the expensive nucleotide sugar precursors, narrow substrate specificity, and low enzyme availability. Comparatively, cellulases or β-glucanases are more abundant, commercially available, and exhibit a broad acceptor-substrate specificity with simple substrates [10, 11]. Biochemical characterizations of cellulases require intensive efforts but are likely to generate new opportunities for the use of renewable resource as biofuels, food and drug additives, and other agricultural products [6–12].

Metagenomic approach as a molecular biological access to retrieve uncultured microbial resource has been widely employed to isolate novel genes and prospect for important biocatalysts [13]. Under the development of metagenomic technology, considerable functional bioenzymes have been isolated from uncultured microorganisms in soils and other environmental samples. The cellulases are one of the most important group of functional bioenzymes targeted by metagenomic technology [10, 13–16].

The need for more suitable biocatalysts has reinforced the scientific research for novel cellulase, especially from the uncultured microbial resources. In this study, a metagenomic fosmid library was constructed from a paddy soil and a novel β-glucanase Umcel9y-1 was successfully screened. The full length gene of Umcel9y-1 was cloned and overexpressed in *Escherichia coli* BL21 (DE3). Biological study revealed that Umcel9y-1 was an efficient endoglucanase with versatile activities (i.e., exoglucanase and transglycosylation), and the potential industrial values of Umcel9y-1 were evaluated.

## Methods

### Sample collection and metagenomic library construction

Paddy soil was collected from Liaoning province (41°07′03”N 122°03′09”E) of China in October 2010. The total microbial DNA was extracted using SoilMaster™ DNA Extraction Kit (Epicentre, Madison, WI) according to the manufacturer instructions. The metagenomic library was constructed using CopyControl™ Fosmid Library Production Kit (Epicentre, Madison, WI) according to the manufacturer’s instructions. DNA products were examined by agarose electrophoresis, and the fragments within the sizes of 25–35 kb were recovered for an end-repair reaction, and ligated into pEpiFOS-1 fosmid vector prepared in the kit. In vitro packaging was performed with a MaxPlax lambda packaging extract kit (Epicentre, Madison, WI). Finally, the products were infected into *E. coli* EPI 300 (Epicentre, Madison, WI). The quality of the library was tested by *Not*I (Promega, Madison, WI) digestion of the prepared fosmids plasmids, and colony counting was carried out by automatic bacteria counter (Shineso Science & Technology Co., LTD, China).

### Cellulase screening and gene cloning

The metagenomic library was screened using the substrate carboxymethylcellulose (CMC, Sigma, St. Louis, MO, USA) for CMC hydrolysis genes. The transformed *E. coli* EPI 300 was inoculated on CMCase screening agar containing 1.0 % tryptone, 0.5 % yeast extract, 1.0 % NaCl, 0.5 % CMC, 100 µg/mL chloramphenicol, and 0.2 mM isopropy-β-D-thiogalactoside (IPTG). The screening agar was incubated at 37 °C for 24 h. After incubation, the plates were stained with 0.2 % Congo-red for 20 min [17]. The CMCase positive clones were screened out by a hydrolysis zone around the bacterial colony. CMCase positive plasmids were enriched with fosmid autoinduction solution and purified by FosmidMAX™ DNA purification Kit (Epicentre, Madison, WI). For subcloning, the plasmid DNA of CMCase positive clone was digested by *Not*I, then recovered for further digestion by *Sau*3AI (Promega, Madison, WI), and ligated into a pBlueScript SK(+) vector (Stratagene, La Jolla, CA). The subclone of CMCase positive transformant was screened further, and the insert DNA sequencing was performed on an Applied Biosystems DNA sequencer, model ABI PRISM 377 (Invitrogen, Shanghai).

### Sequence analysis and secondary structure prediction

Nucleotide sequence translation, and the possible ORF, signal peptide, theoretical pI, and Mw predictions were performed at online programs (ExPASy and SignalP 3.0) [18, 19]. The conserved region analysis and comparison of sequence identity to the related cellulase genes were performed by BlastX (http://www.ncbi.nlm.nih.gov). Sequences of complete cellulase proteins retrieved from the GenBank database were aligned using the CLUSTAL*_*X software and the alignments were corrected manually [20]. Phylogenetic analysis was performed by neighbor-joining algorithm using MEGA v5.0, and the topology of the phylogenetic tree was assessed by the bootstrap analysis based on 1000 replications. The circular dichroism (CD) spectroscopy was carried out to estimate the secondary structure of the purified recombinant protein. The CD spectra were determined at room temperature with a JASCO J-810 spectrometer (JASCO Japan), and the protein concentration was 10 µM in Tris–HCl buffer [21]. The secondary structure of the protein was predicted by the online program Protein Homology/analog Y Recognition Engine v2.0 and the online tool K2D2 [21, 22].

### *Umcel9y-1* gene amplification, overexpression, and purification

*Umcel9y-1* gene of the screened subclone was amplified by PCR using the LA PCR™ Kit Ver.2.1 (Takara, Dalian). A primer pair of 5′-GACACCCATGGGCAGCAGCCATCATCATCATCATCAC-3′ (forward primer) and 5′-GTGTCCATATGTCACATTGTTGGAAGCAA-3′ (reverse primer) was employed in the PCR amplification, and the restriction sites of *NcoI* and *NdeI* were introduced and underlined. The PCR conditions were 1 min at 94 °C, followed by 30 cycles of 10 s at 95 °C, 35 s at 58 °C, and 5 min at 72 °C. The PCR fragments was first ligated into pUC57 (Sangon, Shanghai), then excised by *NcoI* and *NdeI*, ligated into pET-15b^(+)^ vector (Novagen, San Diego, CA), and transformed into the *E. coli* BL21 CodonPlus™ (DE3) strain. The transformant cells were incubated in Luria-Bertani medium (containing 50.0 µg/mL kanamycin) at 15, 22, and 28 °C for appropriate temperature assay. Until the cell density of OD_600_ reached 0.6, the broth was induced by adding 0.1 mM IPTG and followed by additional 5 h incubation. His-tagged recombinant protein in cell disruption (precipitant) was purified by affinity chromatography with nickel-nitrilotriacetic acid agarose resin (Ni–NTA, Qiagen, CA). After sample loading, His-tagged recombinant protein was purified by washing buffer (2 M Urea, 50 mM Tris, 2 mM DTT, 10.0–50.0 mM imidazole, pH 8.0), and collected by elusion buffer (2 M Urea, 50 mM Tris, 2 mM DTT, 500 mM imidazole, and pH 8.0). Then, the purified protein was de-His-tagged by TEV protease at 37 °C. The de-His-tag protein was further purified by affinity chromatography to remove the hydrolyzed His-tags, and the Ni^2+^ ion from Ni-NTA resin was eliminated by dialysis. The purity of recombinant protein was determined by SDS-PAGE using ChemiDoc™ XRS+ system (BioRad, CA), and the protein concentration was estimated by the solution absorbance at 280 nm using a molar extinction coefficient [23].

### Substrate specificity and kinetic analysis

Cellulase activity was assessed by measuring the amount of reducing sugars released from CMC (or other substrates) at 575 nm using dinitrosalicylic acid (DNS) reagent [12]. Enzymatic reaction was carried out in 2 mL of mixtures (in phosphate buffer, pH 7.0) containing 1.2 mL cellulase solution (0.12 mg/mL) and 0.8 mL 2.5 % CMC (w/v) at optimum temperature for 30 min. One unit (U) of hydrolysis activity was defined as the amount of enzyme to release 1 µmol of reducing sugar per minute. The substrate specificity of recombinant enzyme was assayed according to the standard methods in phosphate buffer (pH 7.0) at 37 °C [15, 16], and all of the substrates were obtained from Sigma-Aldrich. Other than CMC, the polysaccharides of hydroxyethyl cellulose (HEC), laminarin from *Eisenia bicyclis*, β-D-glucan from barley, xylan from oat spelt and microcrystalline cellulose (MCC), the cellooligosaccharides of cellobiose (G2), cellotriose (G3), cellotetraose (G4), cellopentaose (G5) and cellohexose (G6), and the aryl-β-glycosides of p-nitrophenol-β-D-cellobioside (pNPCel), p-nitrophenyl-β-D-glucopyranoside (pNPGlc), p-nitrophenyl-β-D-galactopyranoside (pNPGal), p-nitrophenyl-β-D-xylopyranoside (pNPXyl), and p-nitrophenyl-β-D-fucopyranoside (pNPFuc) were selected and determined for substrate specificity. The substrate specificity was assayed under the optimal conditions at a final concentration of 1.0 % substrate (w/v) for 120 min (except 8 h for MCC and 15 min for barley glucan). The specific activities were determined under the optimal condition for 30 min at the substrate concentrations of 40.0 mg/mL for CMC; 20.0 mg/mL for xylan, pNPXyl, pNPGlc, pNPCel, and laminarin; and 15.0 mg/mL for HEC and oligosaccharides. The specific activity of barley glucan was assayed under the optimal condition for 15 min at the substrate concentration of 10.0 mg/mL, and MCC was assayed under the optimal condition for 4 h at the substrate concentration of 20.0 mg/mL. To determine the kinetic parameters of recombinant enzyme, reactions were carried out under the optimal condition for 30 min (except 15 min for barley glucan) at appropriate substrate concentrations (4.0–20.0 mg/mL for CMC, HEC, pNPCel, laminarin and oligosaccharides, and 1.0–10.0 mg/mL for β-D-glucan). The reaction rate versus the substrate concentration was plotted, and the kinetic constants *K*_*m*_ and *K*_*cat*_ were calculated by a nonlinear regression of the Michaelis-Menten equation with GraphPad PRISM version 5.0 (GraphPad Software, La Jolla, CA).

### Biological characterization of recombinant Umcel9y-1

The pH range was determined with appropriate buffers (Citrate buffer, pH 3.0–6.0; Phosphate buffer, pH 6.0–8.0; Tris-HCl buffer, pH 8.0–9.0; and glycine-NaOH buffer, pH 9.0–10.0), and the temperature range was measured from 20 to 90 °C at the interval of 10 °C using the method for enzymatic activity assay [15]. The enzymatic stability at various temperatures and pH were performed as described previously. The recombinant Umcel9y-1 was incubated at various temperatures for 0–90 min and at various pH for 0–96 h, then the residual activities were determined [24]. Effects of metal ions, EDTA, and chemical agents on the enzymatic activity were tested by appropriate concentrations. The final concentration of the divalent metal ions and EDTA were 1.0 mM, respectively, and the concentration of each chemical agent for the reaction mixtures was 10 % (v/v). The halotolerance was determined by measuring residual activity under optimal condition following pre-incubation of recombinant Umcel9y-1 in 1.0–4.0 M NaCl or KCl for 10 days. The enzymatic activity of recombinant Umcel9y-1 in high-salt concentrations was determined by the enzymatic activity assay with a final concentration of 1–4 M NaCl or KCl in reaction buffers [15]. Influences of monosaccharides on recombinant Umcel9y-1 activity (kinetic parameters) toward barley glucan and cellooligosaccharide were evaluated as previously with 100 mM additive to substrate barley glucan, and 200 or 1000 mM additive to cellopentaose [10].

### Substrate hydrolysis products and transglycosylation assay

The hydrolysis products of barley glucan and cellooligosaccharides (G6) by recombinant Umcel9y-1 were detected by high-performance liquid chromatography (HPLC) as described previously [10, 25]. Briefly, hydrolysis was carried out in 1 mL of mixture (in phosphate buffer, pH 7.0) containing 0.6 mL cellulase solution (0.12 mg/mL) and 0.4 mL substrate (50 mg/mL). The hydrolysis was carried out for 24 h on barley glucan and 30 h on G6 at 37 °C. Transglycosylation activity of recombinant Umcel9y-1 was determined in 20 µL mixtures (phosphate buffer, pH 7.0) containing 10 mM pNPGlc, 100 mM glucose, and 0.05 µg recombinant protein. Other than the standard mixture, the reaction system contain 1000 mM glucose was further evaluated. Transglycosylation products were analyzed using a high-performance anion exchange chromatograph with pulsed amperometric detection (HPAE-PAD; Dionex ICS-3000, Sunnyvale CA) as described as Uchiyama et al. [10].

### Statistical analysis

Unless specified otherwise, all assays in this study were performed in triplicate. Data are presented as mean ± SD. Statistic analyses were assessed by Student’s *t* test. The *p* values <0.05 were considered statistically significant.

## Results

### Fosmid library construction and CMCase screening

A fosmid library constructed using the purified metagenomic DNAs contained approximate 2.5 × 10^4^ clones. The sizes of the inserted DNA fragments from 20 randomly selected clones were 18–33 kb with an average size of 22 kb. Many positive clones exhibiting CMCase activity were screened. One positive clone, designated as WP-2, was selected for further characterization due to its high CMCase activity.

### Sequence analysis of CMCase

**Fig. 1:**
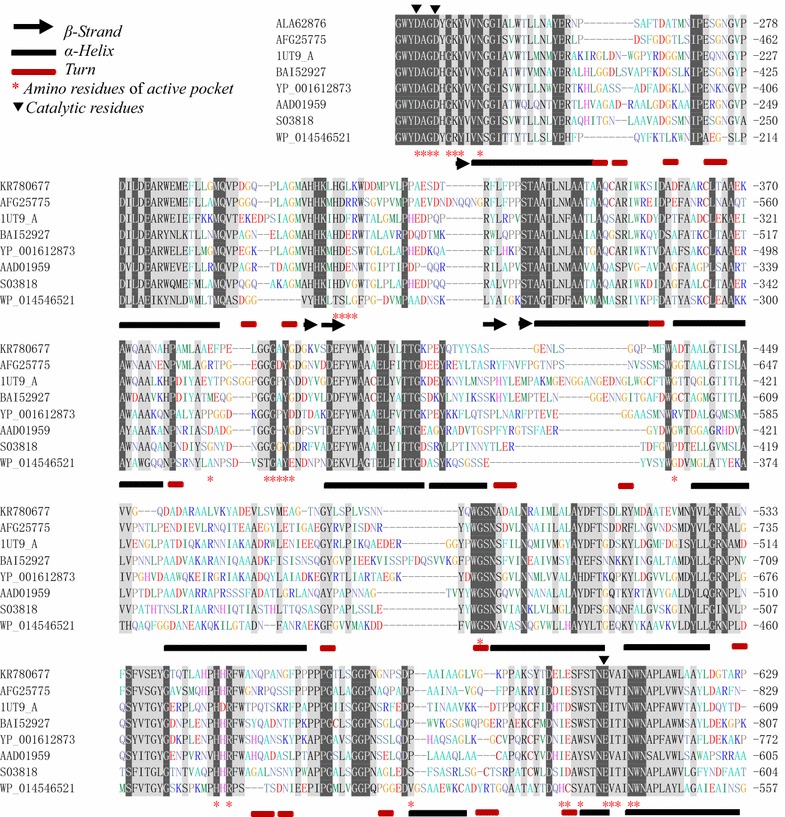
Multiple alignments of Umcel9y-1 with seven highly homologous GHF9 proteins from different microorganisms. The identical residues are shown in *white* with a *black* background, and conservative changes are shown in *black* with a *gray* background. The residues of catalytic site and active pocket, and the secondary structure elements of CbhA from *Clostridium thermocellum* are indicated as the symbols in figure. The amino acid sequences used were as follows: Umcel9y-1 (ALA62876), Cel01 (AFG25775), CbhA (1UT9_A), Man5A (BAI52927), unnamed cellulase (YP_001612873), CelA (AAD01959), unnamed cellulase (S03818), unnamed cellulase (WP_014546521)

The CMCase positive subclone (the recombinant plasmid designated as pumcel9y-1) with an inserted DNA fragment of about 2.7 kb was sequenced and identified by BlastX. A complete gene sequence (2043 bp) of a CMCase was obtained (GenBank accession number: KR780677/ALA62876). The CMCase gene *umcel*9*y*-*1* was expected to encode a protein of 680 amino acid residues (Additional file 1: Figure S1) and was identified as endoglucanase Umcel9y-1 consisting of an immunoglobulin-like domain (Cel-N) at the N-terminal (Amino acids 76–161th: pfam 02927) and an endoglucanase catalytic domain (Amino acids 166–621th: pfam 00759) (Additional file 2: Figure S2). Signal peptide analysis indicated that Umcel9y-1 is a non-secreted protein. The theoretical pI and Mw of Umcel9y-1 were evaluated to be 4.74 and 73296.9 Da, respectively. Phylogenetic analysis of glycosyl hydrolase family 9 (GH9) indicated that Umcel9y-1 was closely (58.4 % amino acids identity) related to the characterized endoglucanase Cel01. Although Umcel9y-1 and Cel01 were the closest related proteins and formed a separated branch within the phylogenetic tree, the two proteins belonged to different themes of GH9 based on their domain organization (Additional file 3: Figure S3). Umcel9y-1 belongs to theme C which contains a family 9 catalytic domain and an immunoglobulin-like domain, but the Cel01 belongs to theme D which has an additional family 4 CBM9 (Additional file 2: Figure S2), and different themes of GH9 proteins indicate different enzymatic activities [25]. The multiple alignment of the amino acid sequence of Umcel9y-1 with highly homologous GH9 enzymes is shown in Fig. 1. The two regions containing putative catalytic residues Asp^234^, Asp^237^, and Glu^607^ are well conserved among GH9 enzymes. The putative catalytic residues Asp^234^ and Asp^237^ act as nucleophile and Glu^607^ is proton donor during substrate hydrolysis [26]. The secondary structure of Umcel9y-1 predicted by the online program Protein Homology/analog Y Recognition Engine v2.0 (Phyre2) contained 29.4 % α-helix and 8.8 % β-strand. However, 23.0 % α-helix and 23.1 % β-strand were obtained from K2D2 database, which calculated by the protein spectrum of circular dichroism spectroscopy (Additional file 4: Figure S4). The distance is large between Umcel9y-1 to the closest spectrum in K2D2 database, suggesting Umcel9y-1 might be a relatively novel protein compare to the database [27].

### Recombinant production of Umcel9y-1

**Fig. 2:**
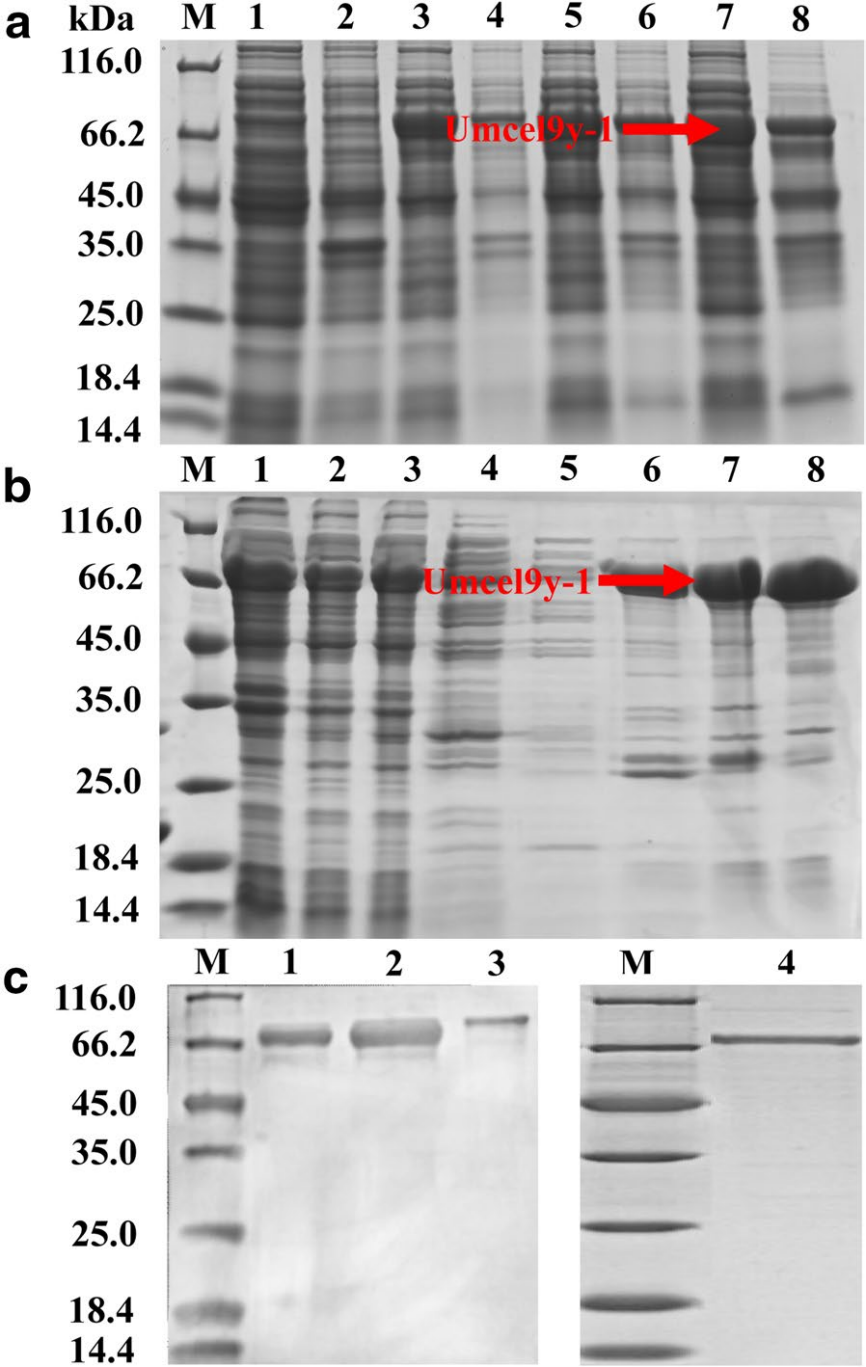
Overexpression and purification of recombinant Umcel9y-1 in *Escherichia coli* BL21(DE3). **a** SDS-PAGE analysis showing the Umcel9y-1 overexpression in *Escherichia coli* BL21(DE3). M, Markers; (1) Control without IPTG at 28 °C (supernatant); (2) Control without IPTG at 28 °C (precipitant); (3) 15 °C overnight (supernatant); (4) 15 °C overnight (precipitant); (5) 22 °C overnight (supernatant); (6) 22 °C overnight (precipitant); (7) 28 °C overnight (supernatant); (8) 28 °C overnight (precipitant). **b** SDS-PAGE analysis showing the purification of recombinant Umcel9y-1 by affinity chromatography. M, Marker; (1) Precipitant before loading; (2) Precipitant flow through; (3) Whole cell disruption before loading; (4) Washing through with 10 mM imidazole; (5) Washing through with 20 mM imidazole; (6) Washing through with 50 mM imidazole; (7–8) Purified eluate with 500 mM imidazole. **c** SDS-PAGE analysis showing the apparent Mw of purified recombinant Umcel9y-1 protein. M, Marker; (1–2) De-His-tag Umcel9y-1; 3, Fusion Umcel9y-1 with His-tag; 4, Repurified de-His-tag Umcel9y-1

His-tagged Umcel9y-1 protein was efficiently expressed as a soluble protein fraction in cells of *E. coli* BL21 CodonPlus™ (DE3) from 15–28 °C. Umcel9y-1 expression production indicated that the optimum overexpression condition in *E. coli* BL21 is at 28 °C with 0.1 mM IPTG (Fig. 2a). His-tagged Umcel9y-1 was purified to almost homogeneity from the soluble protein fraction by affinity chromatography (Fig. 2b). The purified fusion Umcel9y-1 was de-His-tagged by TEV protease, and the recombinant Umcel9y-1 (de-His-tag protein) contained only one additional glycine residue on N-terminal of Umcel9y-1 (Additional file 5: Figure S5). After de-His-tagged, the recombinant Umcel9y-1 was purified again, and the purity was 90.8 % as determined by SDS-PAGE (Fig. 2c). The final yield of the expression was 2.8 mg pure protein per liter of *E. coli* culture, and the purification yield was about 42.5 %. The apparent Mw of recombinant Umcel9y-1 protein was about 70 kDa, and the fusion Umcel9y-1 was little bigger determined by SDS-PAGE (Fig. 2c). The apparent Mws of the recombinant Umcel9y-1 and the fusion protein in SDS-PAGE were consistent with that of prediction at ExPASy online program.

### Hydrolytic properties of recombinant Umcel9y-1

**Fig. 3:**
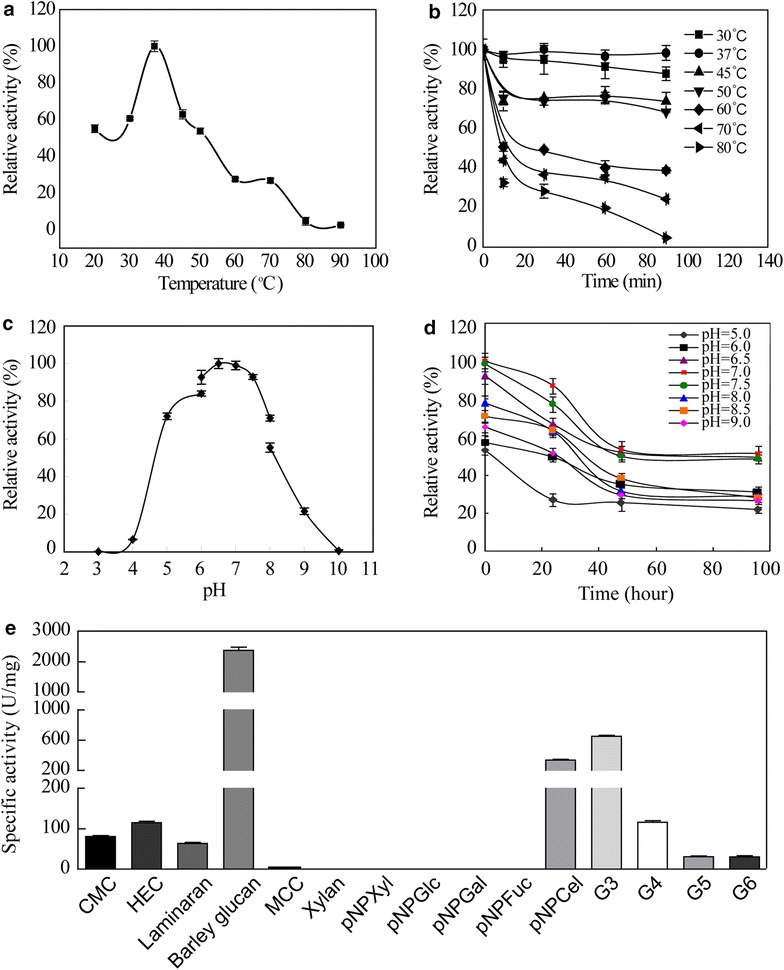
Biochemical characteristics of purified recombinant Umcel9y-1. **a** Temperature range and optimal temperature; **b** Thermal stability; **c** pH range and optimal pH; **d** pH stability; **e** Specific activity for different substrates

Substrate specificity assays showed that CMC, HEC, laminarin, barley glucan, pNPCel, and oligosaccharides (G3-G6) could be hydrolyzed, and MCC was hydrolyzed weakly, but cellobiose, Xylan, pNPXyl, pNPGlc, pNPGal, or pNPFuc was not hydrolyzed. Recombinant Umcel9y-1 exhibited the highest activity toward barley glucan (2372.9 ± 184.3 U/mg) followed by G3, pNPCel, and HEC. The enzymatic activity toward MCC was very low (2.18 ± 0.21 U/mg), and CMC was not the optimal substrate for Umcel9y-1 (Fig. 3e). Substrate specificity assays displayed a wide range of β-(1,4)-, β-cellobiose, β-(1,3)-/β-(1,6)-, and β-(1,3)-/β-(1,4)-/β-(1,6)- linked polysaccharides and amorphous cellulose were hydrolyzed by recombinant Umcel9y-1. Umcel9y-1 exhibits extraordinarily efficient activity on barley glucan and shows high hydrolytic activities to pNPCel and CMC, but no hydrolytic activity was observed for pNPGlc, these results indicate that the enzyme possesses endo-/exoglucanases activities, but not β-glucosidase activity.

The steady-state kinetic parameters of recombinant Umcel9y-1 were determined and compared for the various hydrolysable substrates (Table 1). The enzyme showed the most efficient hydrolytic abilities to pNPCel and barley glucan, followed by cellopentaose, cellotetraose, and cellotriose. Other than barley glucan, HEC was the most hydrolysable polysaccharide for Umcel9y-1. Barley glucan was the optimal polysaccharide substrate and cellopentaose was the optimal cellooligosaccharides substrate. The substrate pNPCel was the only efficiently degraded aryl-β-glycosides in this assays, indicating high exoglucanases activity. The steady-state kinetic parameters further proved that CMC is not the optimal substrate for Umcel9y-1. The *V*_max_ value of recombinant Umcel9y-1 for CMC was also much lower than that of barley glucan. Comparing to other characterized endoglucanases from organisms and environmental samples, Umcel9y-1 showed prominent catalytic efficiency for barley glucan [15, 16, 24, 25, 28, 29].

**Table 1:** Steady-state kinetic constants of the recombinant Umcel9y-1 for various substrates

Substrate	Glycosyl Linkage	*K*_m_ (mM)	*K*_cat_ (s^−1^)	*K*_cat_/*K*_m_ (s^−1^ mM^−1^)	*V*_max_ (μmolmin^−1^ mg^−1^)
HEC	β (1,4)	28.264 ± 1.649	185.650 ± 5.085	6.569	158.730 ± 5.591
CMC	β (1,4)	20.854 ± 1.198	49.212 ± 1.170	2.360	42.191 ± 2.180
Laminarin	β (1,3)/β (1,6)	47.300 ± 3.073	127.510 ± 3.842	2.696	109.892 ± 4.351
Barley glucan	β (1,3)/β (1,4)/β (1,6)	13.313 ± 0.782	3276.381 ± 184.275	246.104	2808.303 ± 104.388
pNPCel	β-Cellobiose	1.381 ± 0.259	466.664 ± 26.168	337.915	400.003 ± 15.661
Cellotriose	β (1,4)	151.673 ± 13.005	897.444 ± 46.260	5.917	769.231 ± 22.347
Cellotetraose	β (1,4)	18.300 ± 1.680	159.820 ± 9.084	8.733	136.990 ± 6.877
Cellopentaose	β (1,4)	0.615 ± 0.097	42.273 ± 4.442	68.732	36.230 ± 3.222
Cellohexose	β (1,4)	5.106 ± 0.888	41.670 ± 3.982	8.161	35.711 ± 4.670

### Biochemical characteristics of recombinant Umcel9y-1

Recombinant Umcel9y-1 showed the maximum activity at 37 °C (Fig. 3a). The temperature stability indicated that Umcel9y-1 was stable from 30–50 °C. More than 90 % original activity was retained after 90 min incubation at 37 °C, and 68.4 % activity was observed after 90 min incubation at 50 °C (Fig. 3b). It was most active at pH range of 5.0–8.0 and the maximum activity was observed on pH 6.5–7.5 (Fig. 3c). When pH was lower than 4.0 or higher than 9.0, the activity was almost lost. The pH stability indicated that recombinant Umcel9y-1 was most stable from 6.5–7.5. Approximately 80 % original activity was retained after 24 h storage at pH 7.0–7.5, and approximately 70 % original activity was observed after 24 h storage at pH 6.5. After 48 h storage at 6.5–7.5, 50 % original activity was retained and the residual activity was stable until 96 h (Fig. 3d).

**Fig. 4:**
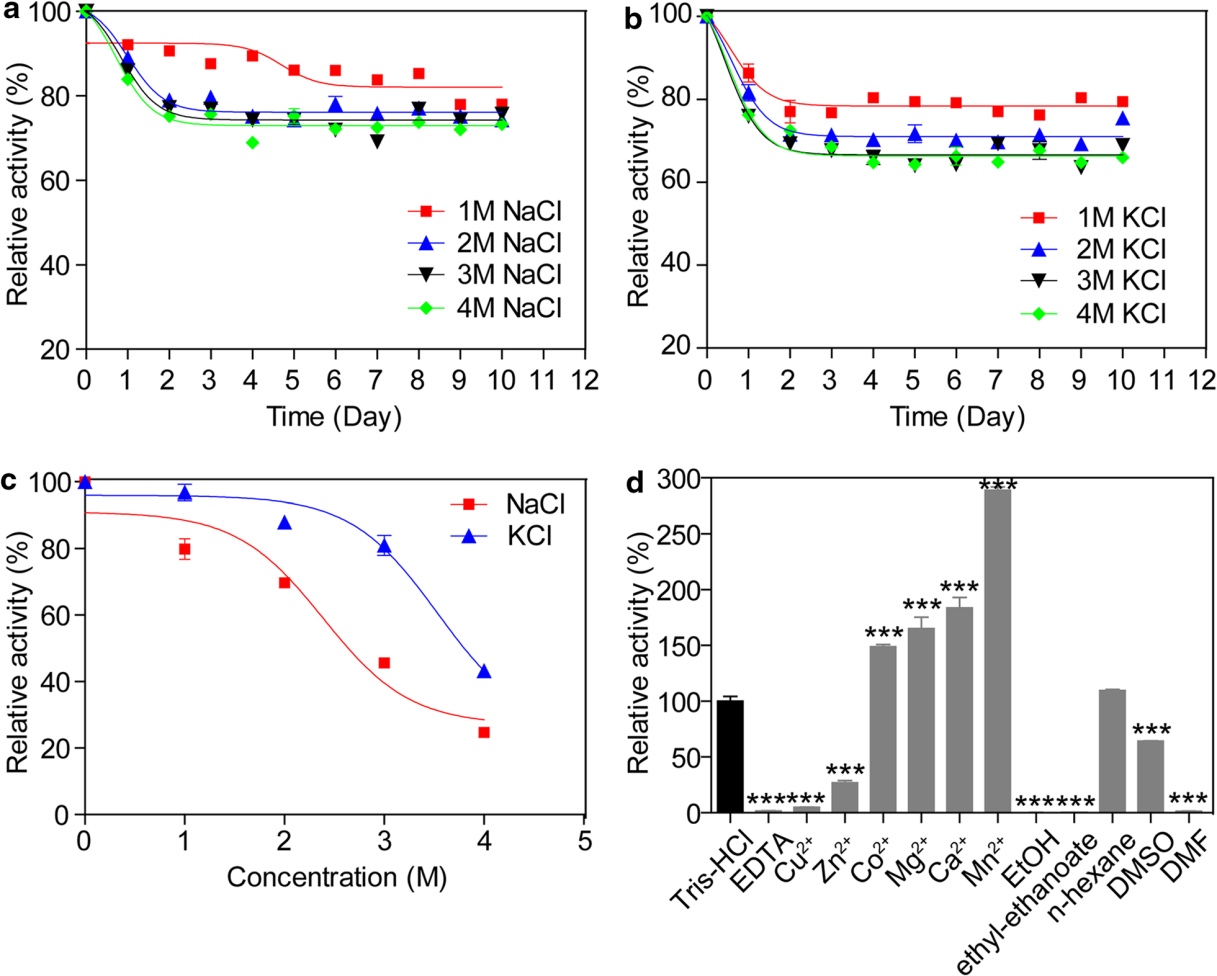
Effects of high-salt concentrations, divalent cations, and chemical agents on enzymatic activity of recombinant Umcel9y-1. **a** NaCl tolerance; **b** KCl tolerance; **c** Catalytic activity in high-salt systems; **d** Divalent cations and chemical agents influences. Data are expressed as mean ± standard deviation. *P < 0.05, **P < 0.01 and ***P < 0.001 versus control (*t* test)

Halotolerance results indicated that Umcel9y-1 was highly salt tolerant, retaining more than 70 % activity to the control after 10 days of pre-incubation with 4 M NaCl or 4 M KCl. More than 80 % activity was observed after 10 days of pre-incubation with 1M NaCl or 1M KCl (Fig. 4a, b). Recombinant Umcel9y-1 was also active in high-salt reaction systems, with 79.9 and 86.2 % activities to control in 1 M NaCl and 1 M KCl, respectively. At concentrations of 2M NaCl or KCl, the relative activities were still higher than 70 % (Fig. 4c). The halotolerance of Umcel9y-1 was even higher than that of the well-known halotolerant protein Cel5G [15].

The activity of recombinant Umcel9y-1 was improved significantly by divalent cations of Mn^2+^, Ca^2+^, Mg^2+^, or Co^2+^ but was intensively inhibited by Zn^2+^, Cu^2+^, or EDTA. The relative activity increased to 288.8 % (Mn^2+^), 183.6 % (Ca^2+^), 165.2 % (Mg^2+^), and 148.8 % (Co^2+^) of the original by 1.0 mM corresponding divalent cation, but the activity was reduced to 26.9 and 5.0 % of the original when 1.0 mM Zn^2+^ and Cu^2+^ was applied, respectively. The enzymatic activity almost disappeared with the addition of 1.0 mM EDTA and was inhibited intensively with the addition of 10 % of ethanol (EtOH), ethyl acetate, N, N-Dimethyl formamide (DMF), or dimethyl sulfoxide (DMSO) (Fig. 4d).

**Fig. 5:**
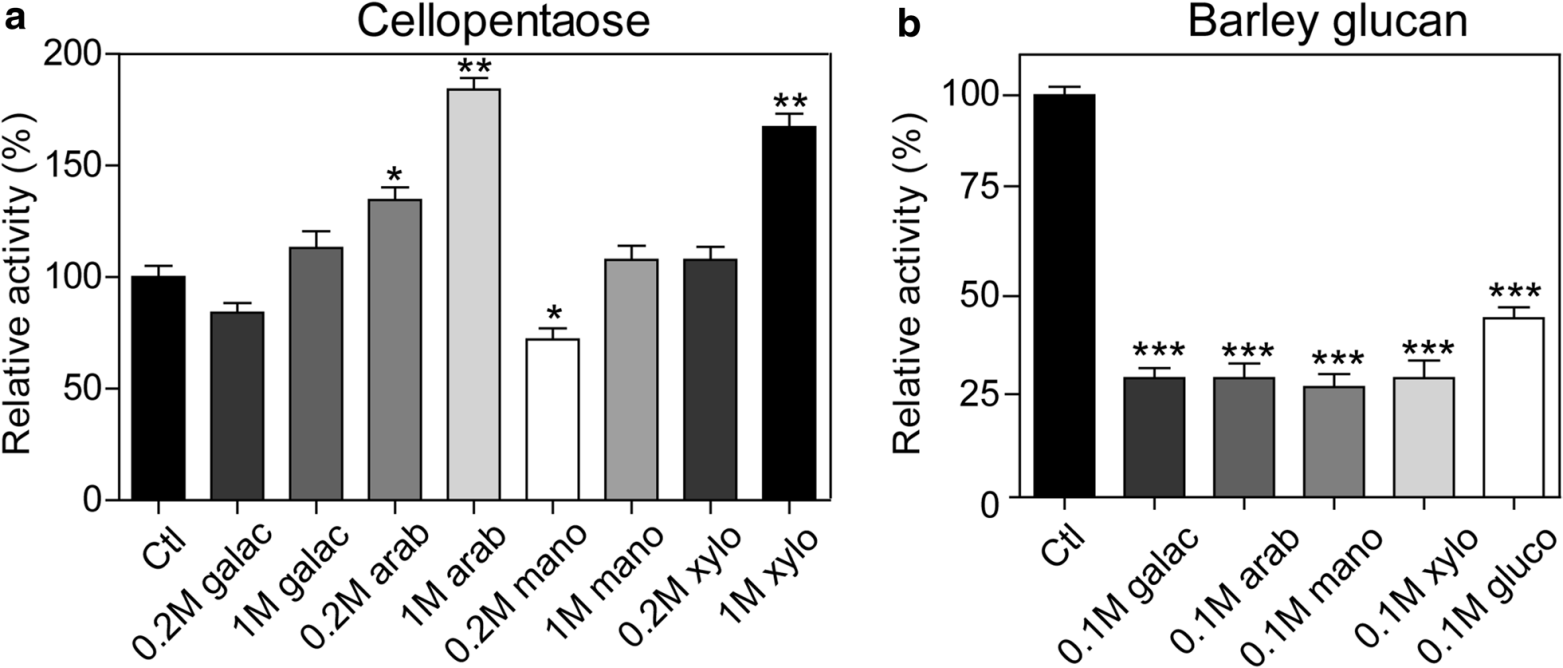
Effects of various monosaccharides on recombinant Umcel9y-1 activity toward barley glucan and cellopentaose. Data are expressed as mean ± standard deviation. *P < 0.05, **P < 0.01 and ***P < 0.001 versus control (*t* test)

The effects of 100 mM monosaccharides on recombinant Umcel9y-1 activity were evaluated for substrate barley glucan, and two concentrations (200 mM and 1000 mM) of monosaccharides were evaluated for substrate cellopentaose. The addition of 100 mM monosaccharides generally decreased *k*_cat_/*K*_*m*_ for barley glucan with exception of D-xylose. The most significant change of *k*_cat_/*K*_*m*_ on barley glucan was observed on 100 mM D-glucose, by which the inhibition was 2.3-fold. However, the addition of monosaccharides generally increased *k*_cat_/*K*_*m*_ for cellopentaose with exception of 200 mM D-galactose, and the degree of stimulation was positively correlated to the concentration of monosaccharide additive. The most significant change of *k*_cat_/*K*_*m*_ on cellopentaose was observed with 1000 mM D-xylose, by which the improvement was 9.5-fold (Table 2). In this study, the Umcel9y-1 hydrolysis activity on barley glucan was exclusively inhibited by 100 mM monosaccharides, and the activity on cellopentaose was significantly increased by 200–1000 mM D-arabinose and 1000 mM D-xylose, but was significantly inhibited by 200 mM D-mannose (Fig. 5). The decrease of hydrolysis activity for barley glucan of this study should be attributed to product inhibition [10], but the monosaccharide stimulations on cellopentaose hydrolysis suggesting that these monosaccharides may induce transglycosylation activity of Umcel9y-1.

**Table 2:** The effects of various monosaccharides on Umcel9y-1 activity toward barley glucan and cellopentaose

Additive	Concentration	*K*_m_ (mM)	*K*_cat_ (s^−1^)	*K*_cat_/*K*_m_ (s^−1^ mM^−1^)	*V*_max_ (μmolmin^−1^ mg^−1^)
Barley glucan					
(No additive)	–	13.700 ± 0.805	3276.382 ± 184.281	239.152	2808.301 ± 104.394
D-galactose	100 mM	5.654 ± 0.995	972.222 ± 68.328	171.953	833.333 ± 35.970
D-arabinose	100 mM	4.404 ± 0.339	972.222 ± 47.514	220.758	833.333 ± 52.142
D-mannitose	100 mM	5.378 ± 0.826	897.442 ± 77.300	166.872	769.233 ± 44.361
D-Xylose	100 mM	3. 706 ± 0.360	972.221 ± 43.778	262.337	833.333 ± 63.100
D-glucose	100 mM	14.039 ± 2.287	1458.334 ± 88.201	103.877	1250.000 ± 59.374
Cellopentaose					
(No additive)	–	0.615 ± 0.097	42.271 ± 4.436	68.732	36.233 ± 3.223
D-galactose	200 mM	0.483 ± 0.060	35.572 ± 4.172	73.644	30.492 ± 2.222
	1000 mM	0.253 ± 0.024	47.810 ± 6.410	188.972	40.980 ± 5.271
D-arabinose	200 mM	1.122 ± 0.133	56.910 ± 6.472	50.722	48.783 ± 4.801
	1000 mM	0.917 ± 0.097	77.783 ± 6.851	84.820	66.672 ± 6.052
D-mannitose	200 mM	0.205 ± 0.036	30.461 ± 3.420	148.585	26.112 ± 2.293
	1000 mM	0.169 ± 0.048	45.571 ± 5.453	269.645	39.064 ± 4.213
D-Xylose	200 mM	0.446 ± 0.060	45.570 ± 4.203	102.175	39.064 ± 3.900
	1000 mM	0.109 ± 0.012	70.710 ± 6.884	648.716	60.612 ± 6.190

### Substrate hydrolysis products and transglycosylation activity

**Fig. 6:**
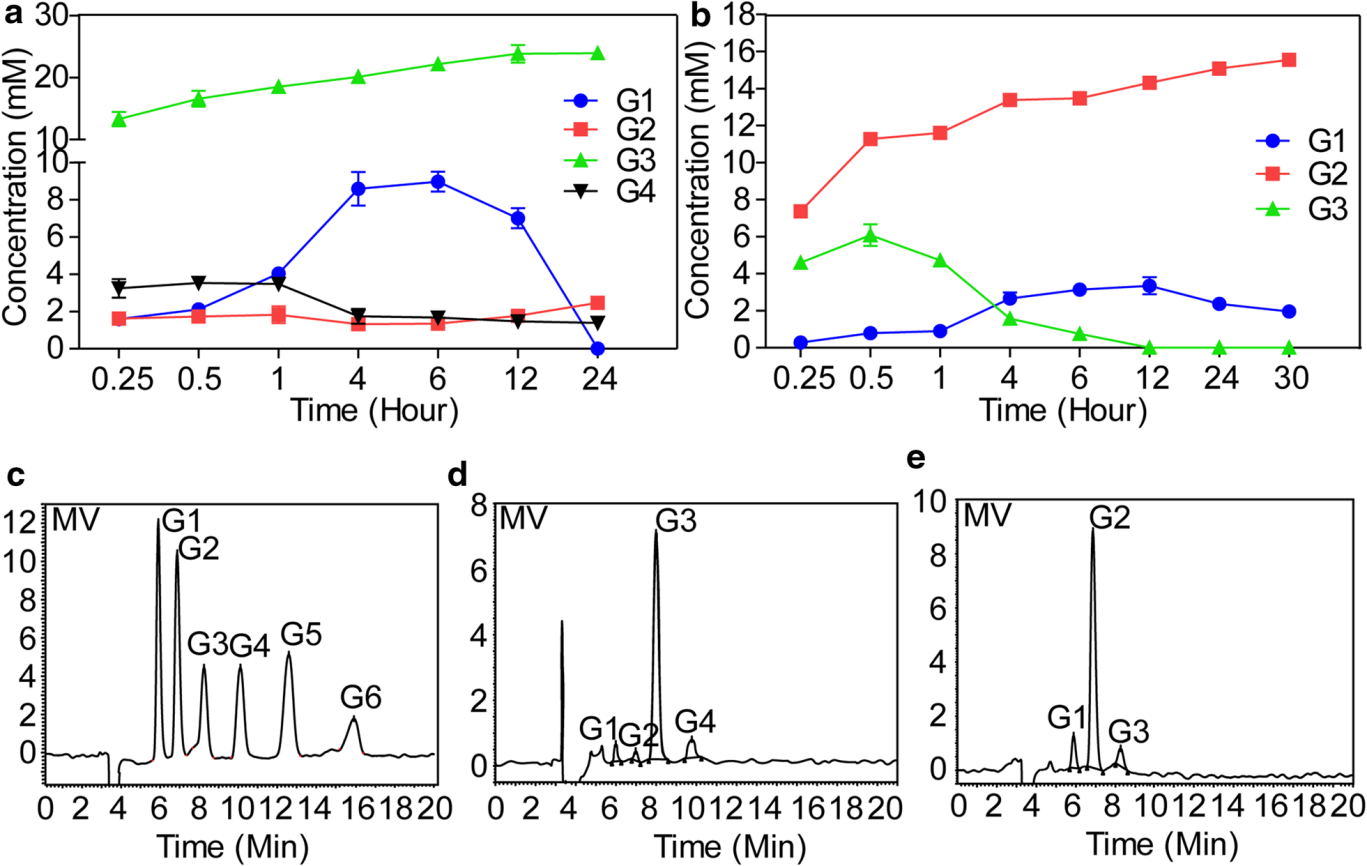
Hydrolysis properties of barley glucan and cellohexose by recombinant Umcel9y-1 monitored by HPLC. **a** Time course of barley glucan hydrolysis; **b** Time course of cellohexose hydrolysis; **c** Chromatogram for G1 to G6 standard mixture; **d** Chromatogram for hydrolysis products of barley glucan; **e** Chromatogram for hydrolysis products of cellohexose

In the polysaccharide and oligosaccharide hydrolysis assays, G3 was the predominant product released from barley glucan, but G2 was predominant product released from G6 by recombinant Umcel9y-1, as determined by HPLC analysis (Fig. 6). Barley glucan hydrolysis initially yielded G4, G3, G2, and D-glucose, but G2 and G4 began to decrease after 1 h and reached steady concentrations at 4 h later; G3 and G1 was continuous increase until 6th h, then D-glucose decreased quickly and almost disappeared at the time point of the 24th h (Fig. 6a). As described above, β-cellobiose (G2) could not be hydrolyzed by Umcel9y-1, and D-glucose is also impossible to be degraded, but the products of G2 and D-glucose were consumed or even disappeared during the hydrolysis course, these results indicated that the transglycosylation may occurred during the hydrolyzation. During the process of G6 hydrolysis, the product G3 was further degraded to G2 and G1, and G3 was disappeared after 12 h incubation. Meanwhile, G1 began to decrease at the time point of 12th h, this also may attributed to transglycosylation (Fig. 6b).

**Fig. 7:**
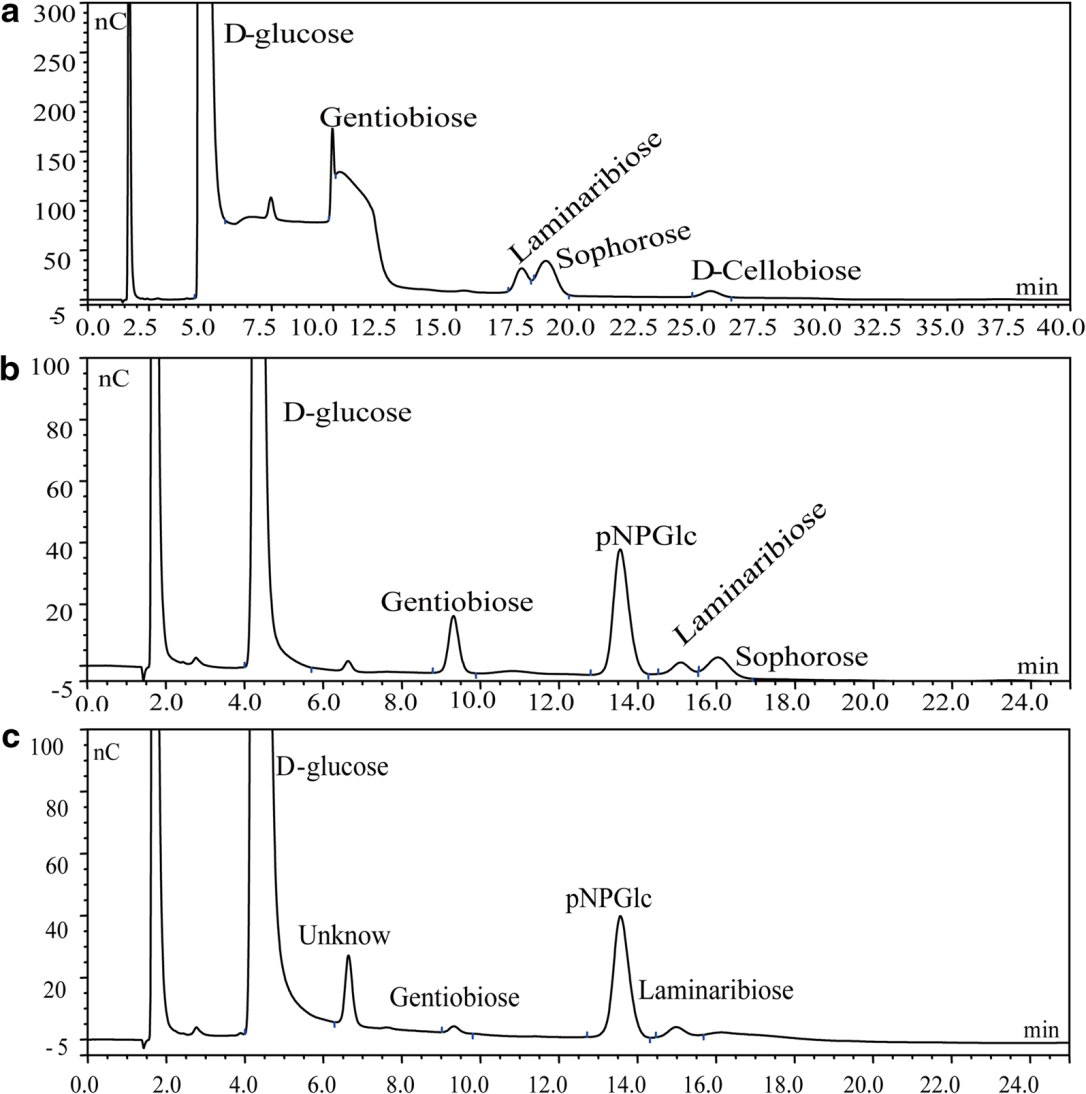
HPAE-PAD analyses of the transglycosylation products of recombinant Umcel9y-1. Chromatograms are given for standard glucodisaccharides (**a**), transglycosylation products with 100 mM D-glucose (**b**), and transglycosylation products with 1000 mM D-glucose (**c**)

Transglycosylation activity was determined using 10 mM pNPGlc and 100 mM (or 1.0 M) D-glucose as substrates. The results indicated that the recombinant Umcel9y-1 with 10 mM pNPGlc plus 100 mM D-glucose resulted in transglycosylation products of gentiobiose laminaribiose and sophorose. When D-glucose concentration elevated to 1.0 M as additive, only gentiobiose and laminaribiose were detected as the products, and sophorose was difficult to be detected by HPAE-PAD at high-D-glucose concentrations (Fig. 7). Transglycosylation product or pNPGlc hydrolysis product pNP was not detected, when recombinant Umcel9y-1 incubated with 10 mM pNPGlc in absence of D-glucose.

## Discussion

The paddy soil is rich of plant biomass (i.e., straw and root) which can be degraded and utilized by cellulose-degrading microorganisms. Unfortunately, only a small fraction of environmental microorganisms (<5 %) can be isolated by laboratory conditions, and thus soil metagenomes represent an unlimited resource for novel biocatalysts exploitation [30]. Metagenomic-derived GH9 glucanase have been isolated from compost and grassland soils [31, 32], aquatic community [33], elephant dung [34], and enrichment culture of alkaline lakes [35], but never from paddy soil. In this study, an efficient and versatile β-glucanase Umcel9y-1 was cloned from the metagenome of paddy soil and characterized for potential industrial development.

Substrate specificity suggested that Umcel9y-1 can hydrolyze CMC and pNPCel that are indicative of endo- and exoglucanase activities, and the enzyme also exhibits transglycosylation activity in presence of D-glucose. Amino acid sequence analysis indicated that Umcel9y-1 belongs to theme C of GH9 (Fig. 1, Figure S2), but phylogenetic analysis showed Umcel9y-1 was separated from theme C and showed closest relatedness to theme D (Additional file 3: Figure S3) [25]. Therefore, the specific modular structure may be one of the key factor for the efficient and multiple activities. Analyses of the X-ray crystal structure of Umcel9y-1 are currently under way. The resulting three-dimensional model may provide clues for glucosyl-binding subsites and offer an explanation for the efficient endo-/exoglucanase and transglycosylation activity. Umcel9y-1 as the theme C protein of GH9 consisted of a Cel-N-term domain and a GH9 catalytic domain, but without carbohydrate-binding modules (CBMs). However, CBMs in cellulases are considered to be responsible for the association of catalytic modules to insoluble carbohydrates. So, GH9 cellulases of themes B and D consisting of CBMs often exhibited efficient activities toward crystalline cellulose, which was not observed on Umcel9y-1 [25, 36].

Among the tested biochemical characteristics, the prominent advantages of Umcel9y-1 are the halotolerant and thermal stability. It is more halotolerant than other salt-tolerant cellulases from saline environment [14, 15, 33, 35]. The stability and activity of Umcel9y-1 in high-salt concentrations are remarkable for an endoglucanase from non-halophilic environmental community (Fig. 4). Umcel9y-1 was highly active at 20–50 °C and the activity was relatively stable at 50 °C. The optimal temperatures of metagenomic-derived cellulases Cel01, Cel5G, and Cel5A also ranged 45–50 °C with rapid inactivation at 60 °C [14, 15, 31]. The relative activity of Umcel9y-1 was greatly enhanced by addition of Mg^2+^, Mn^2+^, or Ca^2+^ but was inhibited intensively by other divalent cations, chelating agent, or chemical agents. This result indicated that divalent cations (especially Mn^2+^) are required for enzyme activation. However, the divalent cations (e.g., Ca^2+^, Co^2+^, Mg^2+^, and Zn^2+^), chelating agents. or other chemical agents (e.g., EtOH, SDS, and DMSO) showed limited influences on the other endoglucanases (e.g., Cel01, Cel5A, Cel9D, and Cel5G) [14, 15, 25, 31].

Another prominent advantage of Umcel9y-1 is the excellent catalytic efficiency on barley glucan. The *k*_cat_/*K*_m_ value for barley glucan (239.14 s^−1^ mM^−1^) is higher than that of most reported cellulases from bacteria, fungi and metagenomes (Additional File 6: Table S1). For example, the *K*_cat_/*K*_m_ value of CelA obtained from *Alicyclobacillus acidocaldarius* was 2.3 s^−1^ mM^−1^ [24]; the *K*_cat_/*K*_m_ value of Cel9D obtained from *Fibrobacter succinogenes* was 0.0097–22.8 s^−1^ mM^−1^ for different substrates [25]; the *K*_cat_/*K*_m_ values of EngD and EngB-CBD that modified from endoglucanase EngB of *Clostridium cellulovorans* were 25.3–42.1 s^−1^mM^−1^ [28], and the *K*_cat_/*K*_m_ value of a endoglucanase from *Arachniotus citrinus* were 40.0–132.0 s^−1^ mM^−1^ at different temperatures [29]. The high *K*_cat_/*K*_m_ indicate that Umcel9y-1 showed prominent catalytic efficiency for barley glucan hydrolysis than other cellulases from bacteria, actinomycetes, fungi, and metagenomes to their optimal substrates.

Different from the most other glucanases, transglycosylation activity was observed for Umcel9y-1 during polysaccharide and oligosaccharide hydrolysis. In the hydrolysis process of barley glucan and cellohexose, the end product of D-glucose was consumed as substrate of transglycosylation reactions to produce G3 and G2, respectively. Other than the minor components of G2 and G4, G3 is the unique predominant hydrolysis product from barley glucan, and Umcel9y-1 might be considered as a potent biocatalyst for G3 production. Moreover, transglycosylation activity can also relieve the product inhibition of cellulases [10], and some monosaccharides even improved the hydrolysis efficiency of Umcel9y-1 toward cellopentaose, but the activation results were not observed for barley glucan.

## Conclusions

In this study, an efficient and versatile *β-glucanase* gene was cloned from the metagenome of paddy soil, and its enzymatic activities were characterized for the potential industrial applications (e.g., β-glucan saccharification, G3 production, or as glycosyltransferases substitute). The recombinant Umcel9y-1 possesses some interesting characteristics (e.g., easiness for expression and purification, high halotolerance, and prominent catalytic ability to barley glucan) and efficient activities of exo-gluancase and transglycosylation, indicating that it is a novel metagenome-derived cellulase, a potent candidate for industrial applications (Addditional file 6: Table S1).

## Additional files

10.1186/s13068-016-0449-6 Nucleotide sequence and putative amino acids of the subclone insert DNA fragment of *umcel9y*-*1* gene. The immunoglobulin (Ig)-like domain was underlined and the GH9 catalytic domain was shadowed.
10.1186/s13068-016-0449-6 Module structures of Umcel9y-1 and the two closest GH9 cellulases. Abbreviations: SP, signal peptide; CBM, carbohydrate-binding module; Cel-N-term, cellulase N-terminal module; GH9, family 9 glycoside hydrolase module. The cellulases are Umcel9y-1 from a uncultured bacterium of paddy soil, Cel01 from a uncultured bacterium of grassland soil and an unnamed cellulase from *Sorangium cellulosum* So ce56.
10.1186/s13068-016-0449-6 Neighbor-joining phylogenetic tree based on complete amino acid sequences of endogluancases, showing the phylogenetic position of Umcel9y-1 in GH9 family. Bootstrap values are shown as percentages of 1000 replicates, and only the bootstrap values above 50 % are shown. GenBank accession numbers are shown in parentheses. Bar, 2 substitutions per 10 animo acid positions.
10.1186/s13068-016-0449-6 Circular dichroism spectra of recombinant Umcel9y-1.
10.1186/s13068-016-0449-6 Putative amino acid sequence of recombinant Umcel9y-1 expressed by *Escherichia coli* BL21 in the vector of pET-15b^(+)^. His-tag in front of recombinant Umcel9y-1 was indicated as red font, TEV protease site was underlined, and the recombinant Umcel9y-1 was shadowed, in which one additional amino acid ‘Glycine’ (G with blue font) was included after de-His-tagged.
10.1186/s13068-016-0449-6 Steady-state kinetic constant comparison of Umcel9y-1 and other efficient cellulases.

## Abbreviations

CMC: carboxymethylcellulose
IPTG: isopropy-β-D-thiogalactoside
DNS: dinitrosalicylic acid
HEC: hydroxyethyl cellulose
MCC: microcrystalline cellulose
G2: cellobiose
G3: cellotriose
G4: cellotetraose
G5: cellopentaose
G6: cellohexose
pNPCel: p-nitrophenol-β-D-cellobioside
pNPGlc: p-nitrophenyl-β-D-glucopyranoside
pNPGal: p-nitrophenyl-β-D-galactopyranoside
pNPXyl: p-nitrophenyl-β-D-xylopyranoside
pNPFuc: p-nitrophenyl-β-D-fucopyranoside
HPLC: high-performance liquid chromatography
HPAE-PAD: high-performance anion exchange chromatograph with pulsed amperometric detection
GH9: glycosyl hydrolase family 9
