# Supplementary figures and images for: A novel efficient β-glucanase from a paddy soil microbial metagenome with versatile activities
Source: Biotechnol Biofuels. 2016 Feb 13;9:36. doi: 10.1186/s13068-016-0449-6 (PMC4752780; doi:10.1186/s13068-016-0449-6)

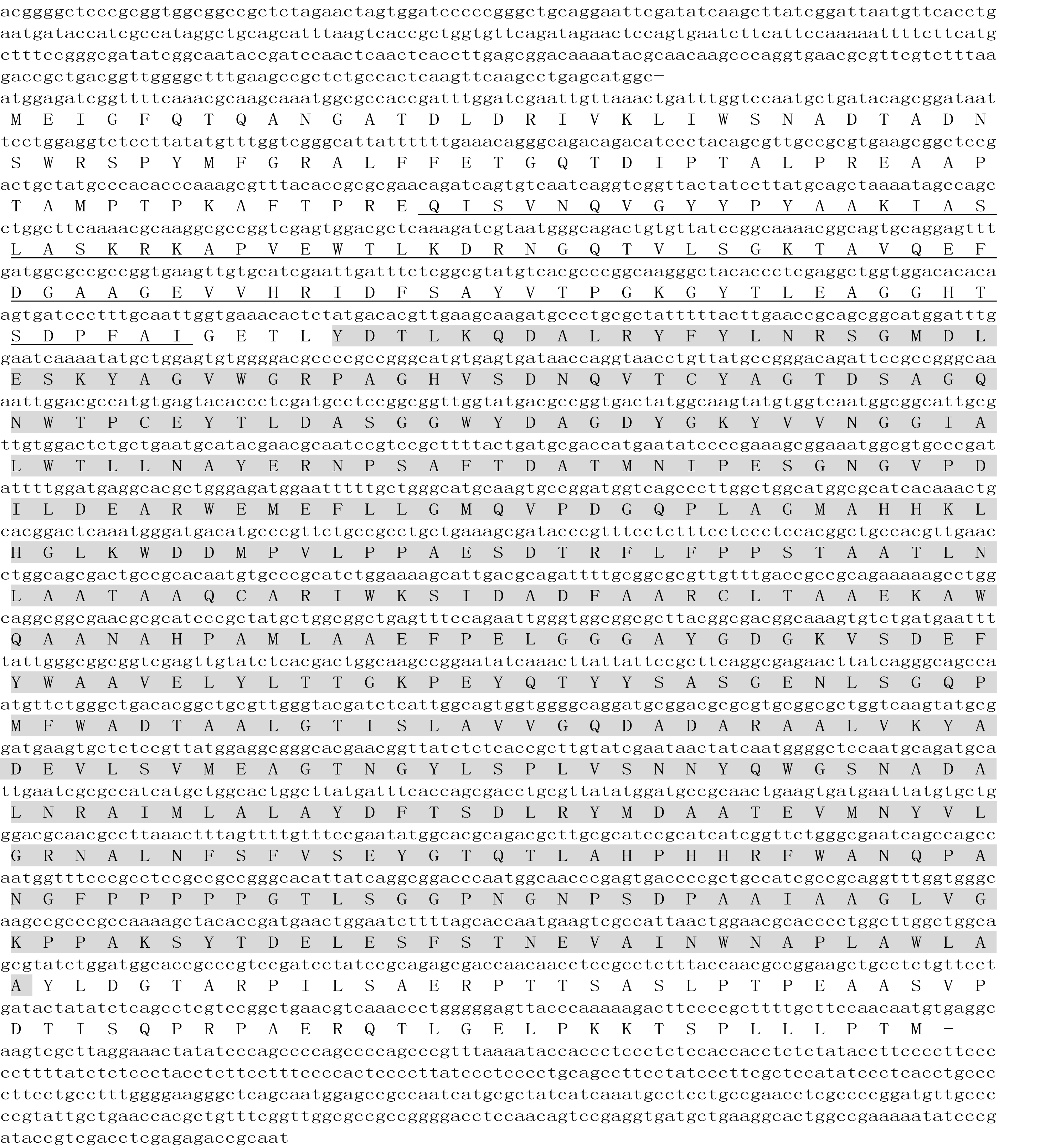

Supplement: Supplementary file 1 — 10.1186/s13068-016-0449-6 Nucleotide sequence and putative amino acids of the subclone insert DNA fragment of umcel9y-1 gene. The immunoglobulin (Ig)-like domain was underlined and the GH9 catalytic domain was shadowed. [file 13068_2016_449_MOESM1_ESM.tif]

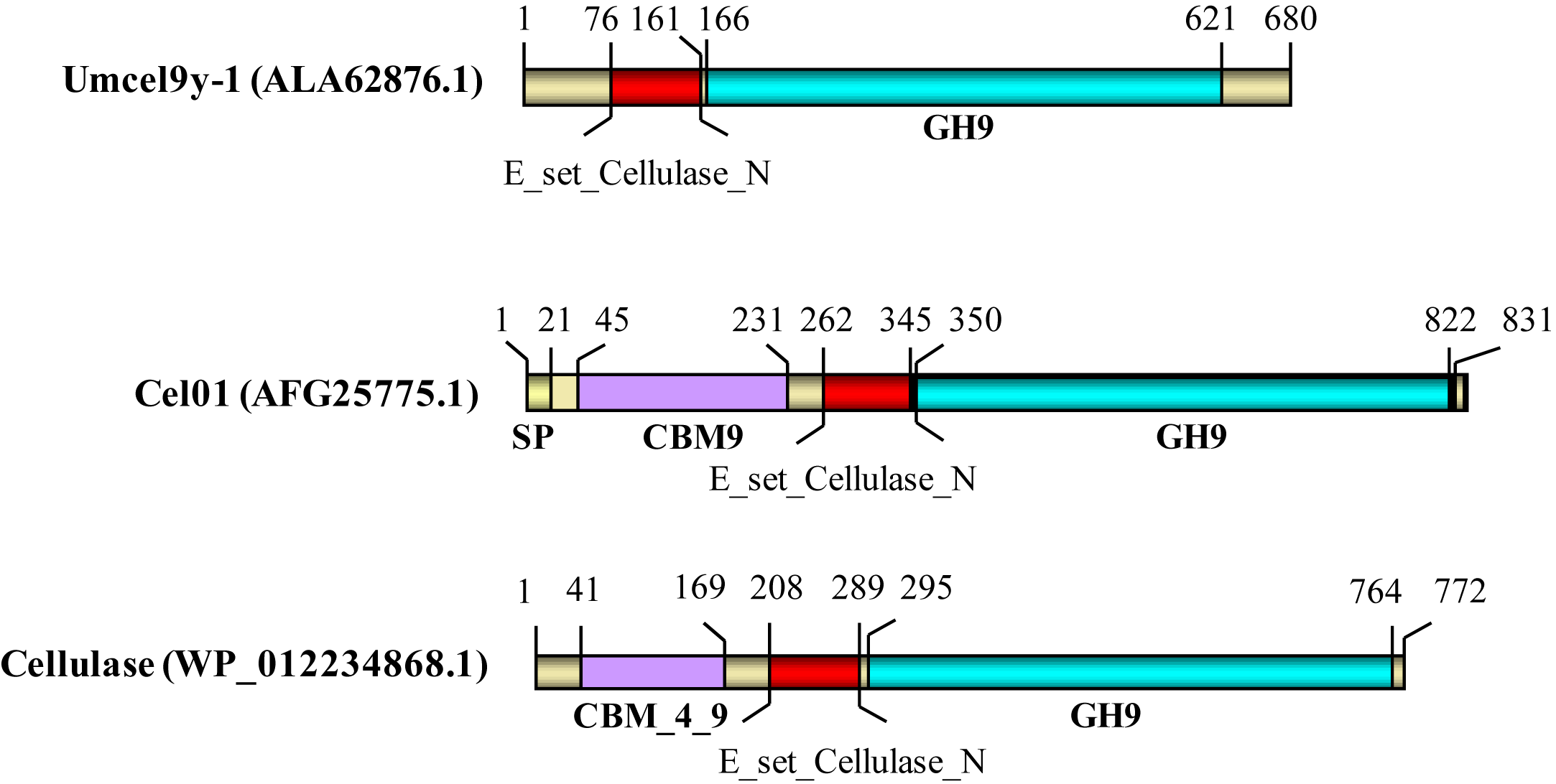

Supplement: Supplementary file 2 — 10.1186/s13068-016-0449-6 Module structures of Umcel9y-1 and the two closest GH9 cellulases. Abbreviations: SP, signal peptide; CBM, carbohydrate-binding module; Cel-N-term, cellulase N-terminal module; GH9, family 9 glycoside hydrolase module. The cellulases are Umcel9y-1 from a uncultured bacterium of paddy soil, Cel01 from a uncultured bacterium of grassland soil and an unnamed cellulase from Sorangium cellulosum So ce56. [file 13068_2016_449_MOESM2_ESM.tif]

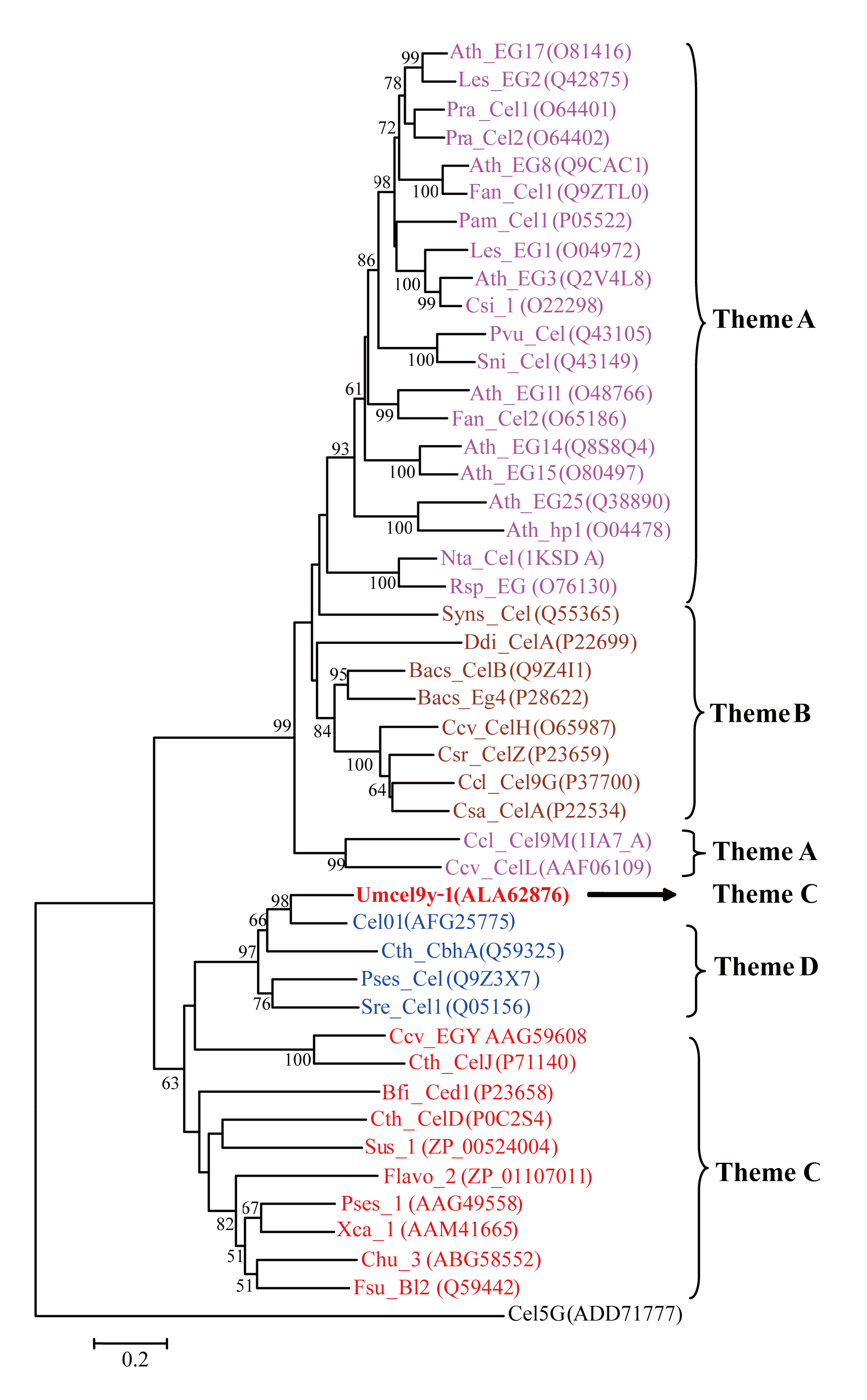

Supplement: Supplementary file 3 — 10.1186/s13068-016-0449-6 Neighbor-joining phylogenetic tree based on complete amino acid sequences of endogluancases, showing the phylogenetic position of Umcel9y-1 in GH9 family. Bootstrap values are shown as percentages of 1000 replicates, and only the bootstrap values above 50 % are shown. GenBank accession numbers are shown in parentheses. Bar, 2 substitutions per 10 animo acid positions. [file 13068_2016_449_MOESM3_ESM.tif]

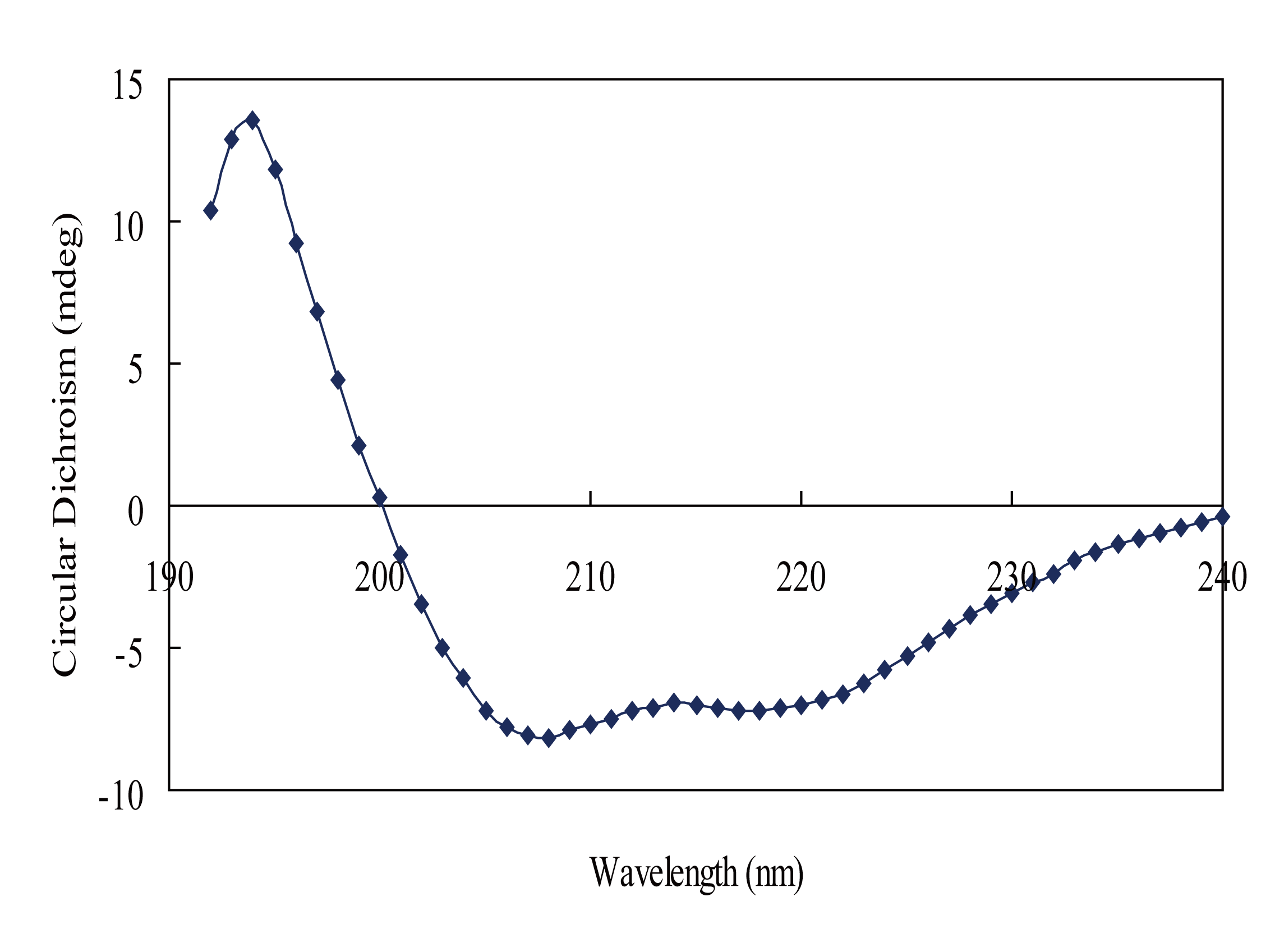

Supplement: Supplementary file 4 — 10.1186/s13068-016-0449-6 Circular dichroism spectra of recombinant Umcel9y-1. [file 13068_2016_449_MOESM4_ESM.tif]

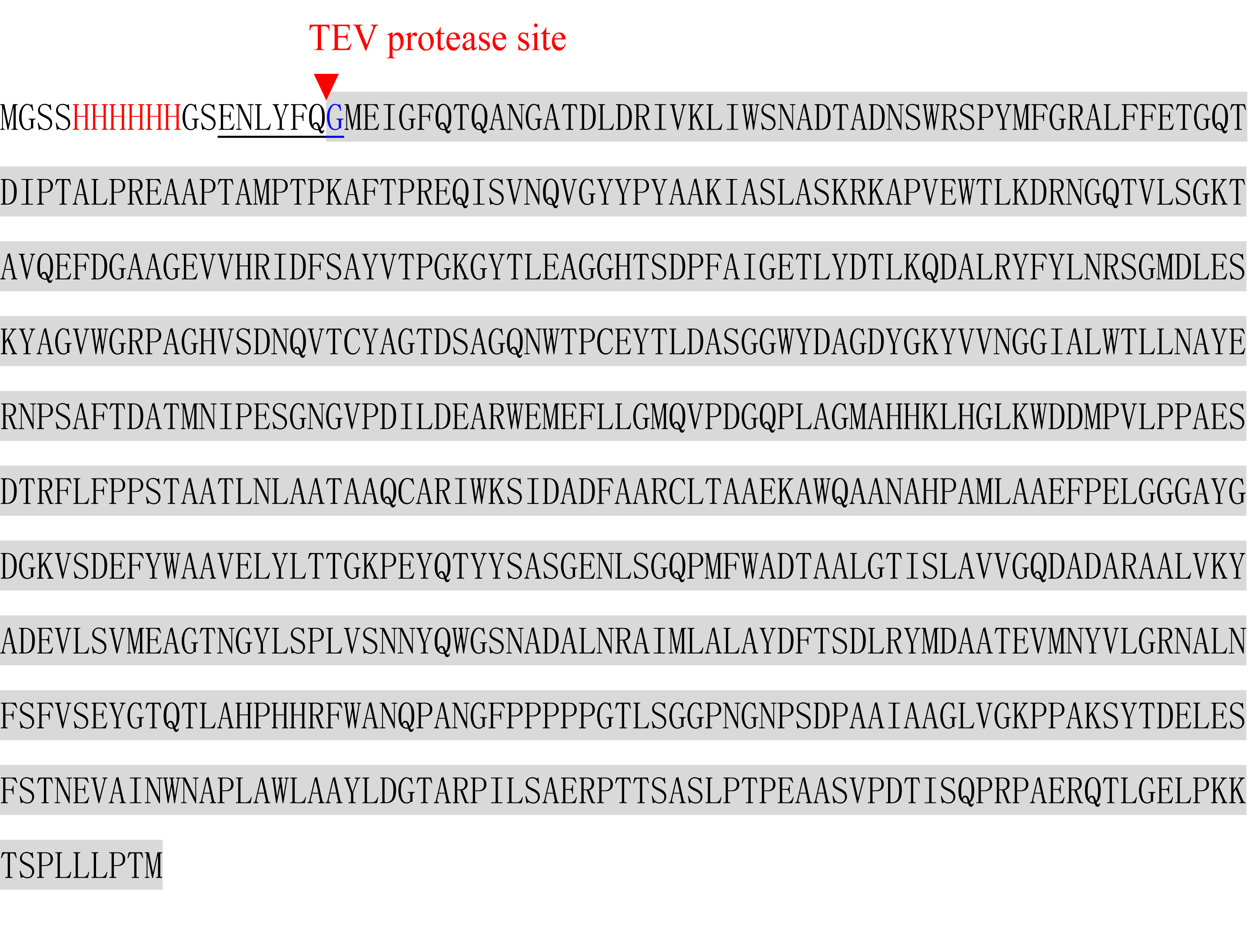

Supplement: Supplementary file 5 — 10.1186/s13068-016-0449-6 Putative amino acid sequence of recombinant Umcel9y-1 expressed by Escherichia coli BL21 in the vector of pET-15b(+). His-tag in front of recombinant Umcel9y-1 was indicated as red font, TEV protease site was underlined, and the recombinant Umcel9y-1 was shadowed, in which one additional amino acid ‘Glycine’ (G with blue font) was included after de-His-tagged. [file 13068_2016_449_MOESM5_ESM.tif]
