# Supplementary material for: A novel efficient β-glucanase from a paddy soil microbial metagenome with versatile activities
Source: Biotechnol Biofuels. 2016 Feb 13;9:36. doi: 10.1186/s13068-016-0449-6 (PMC4752780; doi:10.1186/s13068-016-0449-6)
Supplement: Supplementary file 6 — 10.1186/s13068-016-0449-6 Steady-state kinetic constant comparison of Umcel9y-1 and other efficient cellulases. [file 13068_2016_449_MOESM6_ESM.doc]

**Table S1** Steady-state kinetic constant comparison of Umcel9y-1 and other efficient cellulases

| cellulase | *k*_cat_/*K*_m_  (s^-1^mM^-1^) | Substrate | Original |
| --- | --- | --- | --- |
| Umcel9y-1 | 239.152 | Barley glucan | Metagenome |
| Umcel9y-1 | 337.915 | pNPCel |
| CelA | 2.300 | pNPCel | *Alicyclobacillus acidocaldarius* |
| Cel9D | 0.0097  4.27  15.5  18.5  22.8 | G2  G3  G4  G5  G6 | *Fibrobacter succinogenes* |
| EngD  EngB-CBD | 42.1  25.3 | CMC  CMC | *Clostridium cellulovorans* |
| Unnamed endoglucanase | 40.0-132.0 | CMC | *Arachniotus citrinus* |
| Td2F2 | 30.6  68.8  6.38  1.61  3.35  5.14  0.94  2.27  3.84  0.19  1.14  5.91  0.16  1.53 | pNPGlc  pNPFuc  pNPGal  G2  G3  G4  G5  Laminaribiose  Laminaritriose  Laminaritetraose  Laminaripentaose  Sophorose  Gentiobiose  Lactose | Metagenome |
